# Annotations

**Published:** 1900-11-10

**Authors:** 


### Annotations.

### The Revolt of the Colleges.

The General Medical Council is the supreme body appointed by law to maintain the register and to ensure that no one shall be admitted to its roll unless he or she has undergone a proper course of study. With the object of maintaining the standard of medical education the General Medical Council not only lays down certain conditions as to the curriculum, but visits the examinations held by the different qualifying bodies.

So great are its powers that most of these bodies have accepted, with more or less good grace, such regulations as have been laid down. But the Royal Colleges of Physicians and of Surgeons in London, bodies which have never shown themselves lacking in a full sense of their own importance, have revolted and have had the temerity to refuse to recognise certain regulations laid down by the Council in regard to the first year of study. What would have happened if the Council had dealt firmly with this revolt and had definitely refused to register as medical students those unfortunate young men who proposed to follow the scheme of education laid down by the colleges in defiance of its ruling we cannot tell, although we may hazard the conjecture that the world would have gone on much as usual. But it was not difficult to prophecy what would be the result of the Council allowing itself to be brow-beaten by the Colleges. Anyone could have foreseen that, in the competition for candidates no one College could afford to allow other Colleges to undersell it in the way of facilities for study, and that if the London Colleges were to maintain their right to allow prospective candidates for their diplomas to pass their first year of so-called medical studies at Board Schools, or any outside institution which they chose to recognise as places of study, the other Colleges could not afford to go on demanding from their candidates an honest five years at a medical school. What has happened is just what might have been expected, for the Irish Colleges, seeing how lightly the English Colleges have been dealt with, have joined in the fray, and now announce that they will not be bound by decisions of the Council which other Colleges are allowed to disregard. All this is very hard on the poor students. Those that trust the London Colleges may possibly find themselves refused registration, while, if the wind blows the other way, those who have loyally submitted to the regulations, and have gone in for five years of medical study, may find themselves in competition with others who have got off with only four, which is hardly fair. Who can any longer say that doctors are not men of business, and equal to any smartness when such "cutting" is going on in the diploma trade? The Colleges and the Universities may do what they like as to their higher and purely ornamental diplomas and degrees, but in regard to the qualification by which men are admitted to the Register, it is to be hoped that the General Medical Council will stick to its guns and maintain its right to fix the standard.

### The Birmingham Medical and Surgical Consultative Institution.

This institution, which is intended to provide the advice of a "consulting physician" to working people at a fee of half a guinea, has held its first annual meeting. Judging from the report the success achieved has not been very great. The consulting rooms were opened in May. Since then there have been 222 patients, and the financial report shows a deficiency of £343 13s. 6d., or a loss of rather more than a guinea and a half for every case. The fact is that the Birmingham working man is a good deal sharper than the good people who have set up this consultation institution seem to imagine. He not unnaturally wants his money's worth, and sees no reason why he should pay half a guinea to an institution when there are plenty of private practitioners quite ready to deal at five shillings. The mistake is in thinking that what the working man wants is a "consultation." What he really wants is "better advice," and he measures up the position of the doctor by the status of his patients. This is not in Birmingham alone. Everywhere the poor man believes that the rich man lives on the best, and gets for his own use the best of whatever is going; and when the poor man is ill what he hankers after is not the sort of advice and help which the rich think good enough for the poor, but a slice of the same sort of advice and help that his master gets when he is ill. That is the key to the desire for consultations; and for the sake of getting this "best" advice reputedly poor people are often found to open unexpected hoards. But when they thus open their pockets they are careful enough to get what they pay for. They know perfectly well who are the doctors who attend their wealthy neighbours, and they quite well recognise the impassable gulf which separates these men and their advice from the sort of advice offered by advertising institutions. The first lesson that the managers of the Birmingham Consulting Institution have to learn is that its success must depend on the confidence which the working people repose in the consultants it employs. When this confidence has shown itself by overflowing waiting rooms it will be time enough for them to learn their second lesson, namely that their consultants will find it worth their while to start on their own account. Then, the whole affair will have to be begun over again.

### The Bishop's Pub.

The Bishop of Chester is well known for his practical efforts to reform the drink trade. It is perhaps too soon to say whether he is right or wrong, but there can be no doubt that a reasonable attempt to mend the public-houses is more likely to lead to good results than the frantic efforts to end them which are made by some less judicious reformers. If the drink trade stood on the same footing as any other business, we should hardly venture to anticipate success for the scheme which he advocates so earnestly. But the fact has to be recognised that the sale of intoxicating liquors is a monopoly under the direct control of the justices, and that, with the growth of public opinion in favour of order and decency, this monopoly is likely to fall by preference into the hands of those who can show the greatest decency and order in their conduct of the trade. We do not pretend to say whether the "Bishop's pub" or, to be more precise, the public-houses to be opened by the People's Refreshment House Association, Limited, will succeed, but considering the health interests involved, we may fairly wish the new venture every prosperity. We do not think it will oust the brewers, but we are quite clear that the existence of a thoroughly well-conducted house is an advantage to any district, not merely by way of the facilities it offers to decent people, but by fixing a standard by which the licensing justices may be guided in renewing the licenses of the other houses round about. This object-lesson the "Bishop's pub' ought to give. If it fails to show such an example and to maintain such a standard then it fails indeed; but this is not likely.

